# Spinal adhesive arachnoiditis: three case reports and review of literature

**DOI:** 10.1007/s13760-020-01431-1

**Published:** 2020-08-24

**Authors:** Szymon Jurga, Olga Szymańska-Adamcewicz, Wojciech Wierzchołowski, Emilia Pilchowska-Ujma, Łukasz Urbaniak

**Affiliations:** 1 Department of Neurology, University Hospital, Zielona Góra, Poland; 2 Department of Radiology, University Hospital, Zielona Góra, Poland

**Keywords:** Paraparesis, Adhesive arachnoiditis, Spine MRI, Spine imaging

## Abstract

Spinal adhesive arachnoiditis is a rare pathology involving pia mater of the spinal cord and nerve roots. It can potentially lead to disability—many patients end up wheelchair-bound due to subsequent paraparesis. It is an infrequent but possible cause of lower extremities weakness in patients with a history of spinal surgery, epidural anaesthesia, myelography or spinal tumors. Three patients, one male and two females, admitted to our unit due to paraparesis presented at least one of the above mentioned risk factors. Each of them had a severe course of illness—progressive paresis of lower extremities. All above cases were diagnosed with spinal adhesive arachnoiditis confirmed with Magnetic Resonance Imaging (MRI) scan—the most sensitive and specific diagnostic tool. Despite conservative treatment and intensive rehabilitation none of the presented patients preserved the ability to mobilise independently. Considering spinal adhesive arachnoiditis in patients with paraparesis and history of typical risk factors should be included in clinical diagnostic procedure.

## Introduction

Adhesive arachnoiditis is a rare entity caused by an inflammatory process of pia mater. The symptomatology varies significantly between cases—from asymptomatic, through painful radicular syndromes to severe disability caused by paraplegia. Just like its natural history, aetiology of the disease is also heterogeneous including infections, trauma and spinal tumours. One of the important risk factors of adhesive arachnoiditis is iatrogenic damage caused by neurosurgical interventions, injections of oil-based contrast agents or epidural anaesthesia to name a few. We would like to present three cases of spinal adhesive arachnoiditis admitted to our unit—each of different aetiology and all presenting severe course of disease.

## Case reports

B.S., female, 50 years old, treated at outpatient department (OPD) due to a painful radiculopathy caused by a lumbosacral discopathy (diagnosed with MRI done in February 2017) was subsequently transmitted to Neurology Department in February 2017 due to bilateral lower limb weakness, bladder incontinence and shooting pains radiating to lower extremities—the symptoms worsening over several weeks. She had a history of orthopaedic surgery—bimalleolar fracture and luxation of the right ankle treated with ORIF (open reduction internal fixation). In May 2015 under spinal block (L3–L4) anaesthesia, surgery and early postoperative recovery were all uneventful.

**Fig. 1:**
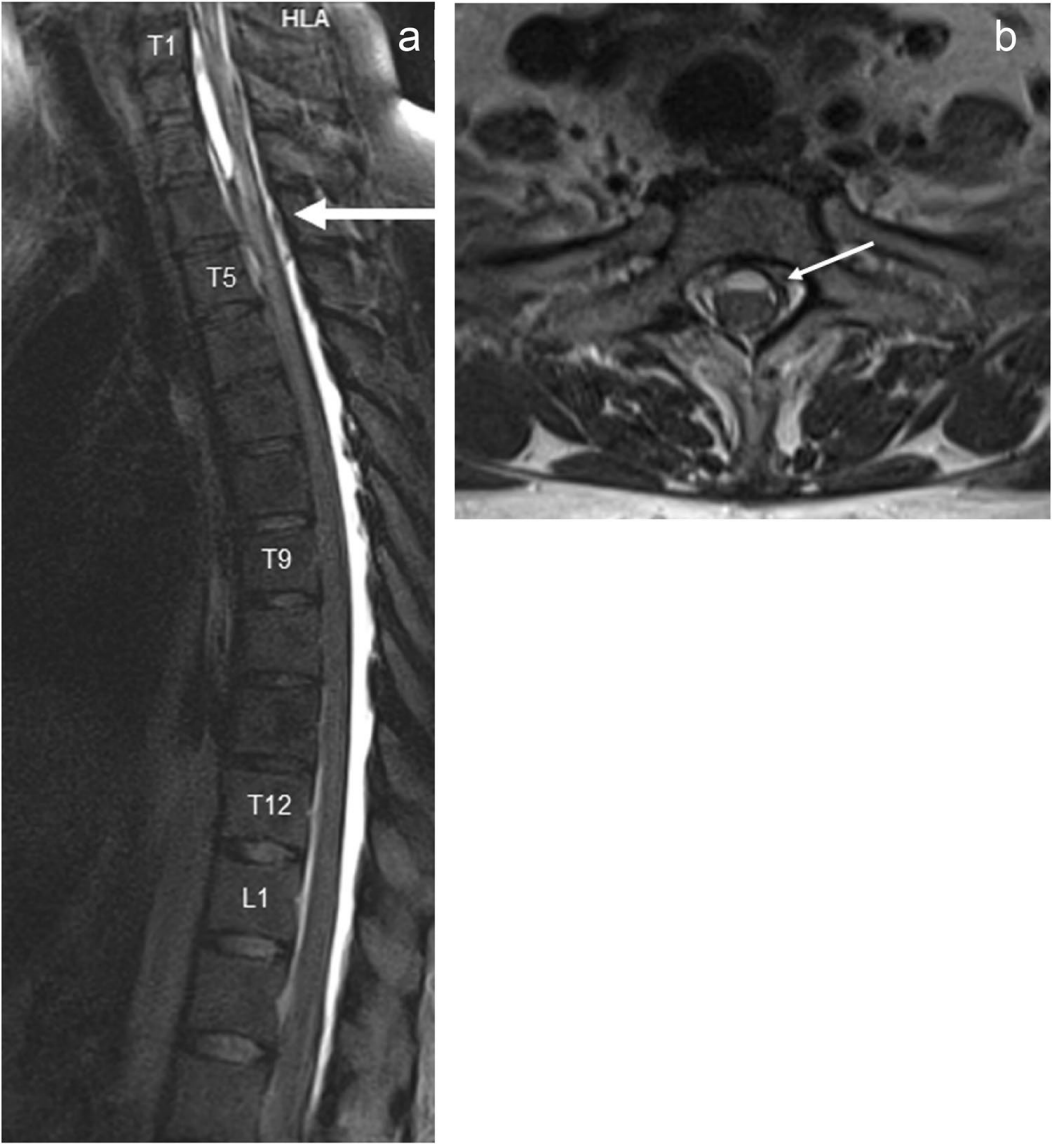
**a** MRI T2-weighted STIR-sagittal view of spine, **b** MRI T2-weighted STIR-transversal view of spine Arachnoid adhesions in spinal canal of the upper thoracic and lower cervical spine. Spinal sac and spinal cord deformities. Strict adhesion of the spinal cord to the front wall of dura mater at the Th2–Th3 level with a coexisting malacia

Neurological examination on admission showed spastic paraparesis with the muscle strength of 2–3 on Lovett scale and decreased superficial sensation at T4–T5 level. The MRI of the thoracic and cervical spine showed spinal cord and thecal sac deformity at the T2–T3 level caused by arachnoid adhesions with a coexisting 4-cm-long lesion of spinal cord extending from the upper edge of the T2 vertebral body down to the upper part of T4 vertebra as well as reduction of perispinal fluid reservoir from T4 down to T9 and from T12 down to L1. Strict adherence of the spinal cord to the spinal canal was noted (Fig. 1). Based on this MRI results a diagnosis of adhesive arachnoiditis was made. The patient did not meet criteria for neurosurgical intervention. She was treated with corticosteroids with no effect. The patient was transferred to Rehab Unit but she presented no improvement of motor function after physiotherapy—on the day of discharge the patient was wheelchair-bound.

**Fig. 2:**
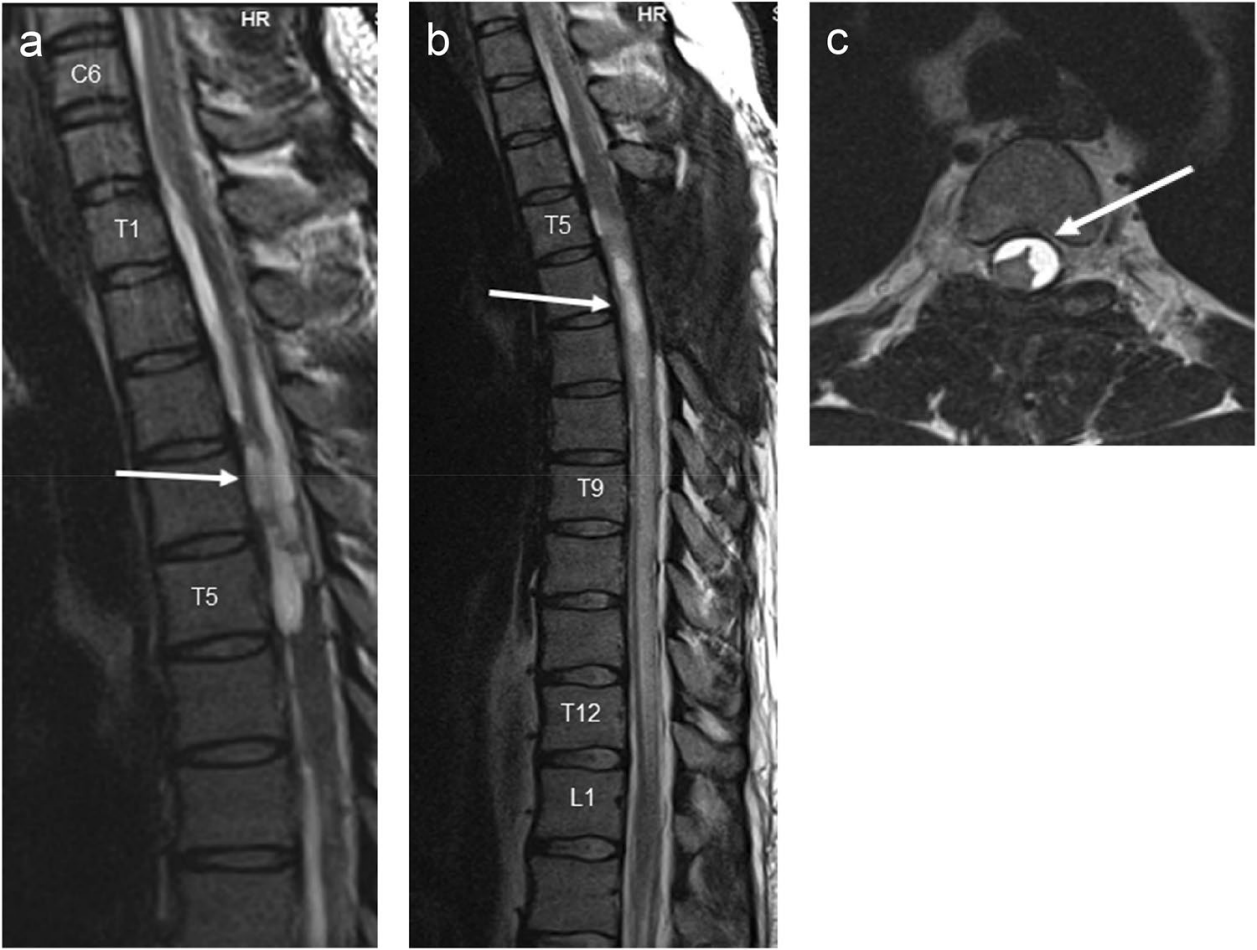
**a** MRI T2-weighted STIR-sagittal view of spine; intrathecal cystic mass at the T1–T5 level with contrast enhancement visible in spinal nerve roots (May 2016), **b** MRI T2-weighted STIR-sagittal view of spine*,* **c **MRI T2-weighted STIR-transversal view of spine; spinal cord malacia extending from half the height of T6 vertebral body down to the T11–T12 (October 2016)

K.M., male, aged 26, with known insulin-dependent diabetes and hypothyroidism, transferred to the Neurosurgery Ward on 5 February 2016 from Medical Ward of another hospital, where he had been treated for abnormal glycemic levels and persistent vomitus. Further diagnostic procedures revealed obstructive hydrocephalus in the course of posterior fossa tumour. During his stay in the Neurosurgery Ward an emergency external ventricular drainage was performed and a tumour resection surgery was scheduled. The excised tumour was histologically evaluated as pilocytic astrocytoma WHO grade I. Three weeks after the surgery the patient was readmitted to the ward due to severe headache and nausea. Subsequent CT scan revealed internal hydrocephalus. Due to its aggravation in the following days the ventriculoperitoneal shunting was performed. After the procedure patient improved clinically and radiologically. On 23rd May 2016 the patient was once more admitted to the Neurosurgery Ward, this time due to increasing lower extremities weakness with coexisting decreased superficial sensation. An MRI scan of the cervical and thoracic spine showed intrathecal cystic mass at the T1–T5 level with a contrast enhancement visible in spinal nerve roots (Fig. 2a). Due to history of expansive process the MRI images were reassessed and based on that a thoracic spine surgery was performed. Intraoperative findings raised a suspicion of inflammation of the spinal cord. The histopathological examination revealed reactive fibrosis. Lower limbs muscular strength decreased in the postoperative period. A neurological review was requested. Steroid therapy was initiated. This led to glycemic dysregulation. The therapy brought no improvement—the patient was transferred to Rehab Unit with a spastic paraplegia. The thoracic spine MRI performed on 20 October 2016 revealed a strict adherence of distorted spinal cord and thecal sac at the T4 level with a concomitant syrinx formation, adhesions between the spinal cord and dura mater from T4 up to T6 level as well as intrathecal cystic lesions between T5 and T6 level. Along with the aforementioned pathology a broad area of spinal cord malacia was shown—extending from half the height of T6 vertebral body down to the T11–T12 borderline (Fig. 2)—thus a diagnosis of chronic adhesive arachnoiditis with spinal cord malacia was made. Patient remained wheelchair-bound despite regular care of physiotherapists. A baclofen pump was implanted with a slight improvement in terms of lower limbs spasticity.

**Fig. 3:**
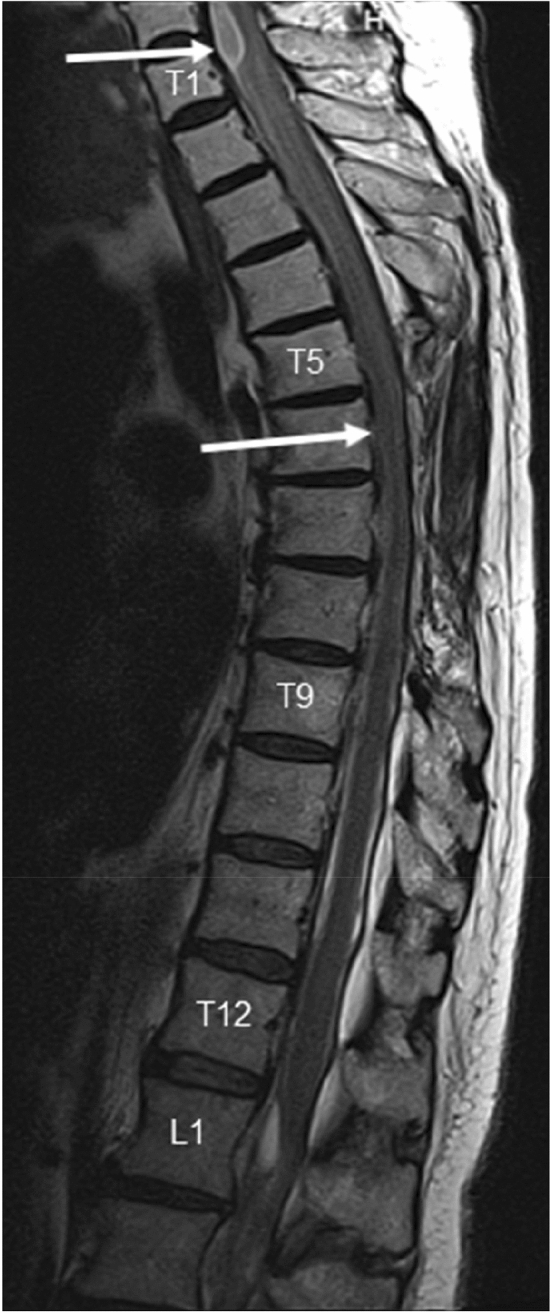
MRI T2-weighted STIR-sagittal view of spine board strict adhesion of the spinal cord to the dura mater in the thoracic spine with complete reduction of front fluid collection of the thecal sac. Numerous adhesions of pia mater with cystic lesions in the front part of the spinal canal at the cervicothoracic and thoracolumbar junction

G.W., female, with a known history of ventriculoperitoneal shunting due to an internal hydrocephalus (18th September 2015), spinal canal tumor resection at the T7–T8 level (benign schwannoma) and a radical right-sided mastectomy due to breast cancer in October 2016. The woman was on systemic cancer treatment consisting of ACP + trastuzumab and hormonal therapy. During her stay at the Oncology Unit of our hospital (May 2017) patient experienced acute weakness of lower extremities. Reviewed by neurologist consultant the evidence of pyramidal paraparesis with bilaterally positive Babiński sign and superficial sensation impairment down from the T4–T5 level was found. A thoracic spine MRI revealed focal ischemic lesions—first of them of about 3 cm of length localised between levels T5 and T6 s and the second one of similar size at the T7–T8 level (benign schwannoma—due to its benign nature no adjuvant chemo or radiotherapy was performed) with a concomitant oedema of the spinal tissue. Also spinal canal presented a multi-level, thecal sac deformity with formation of fluid collections at C7–T1 and T12–L3 levels. The lesions were causing compression and deformation of neural structures in the spinal canal (Fig. 3)—with clinical picture indicating the diagnosis of adhesive arachnoiditis. The chemotherapeutic treatment was modified in aim to reduce its neurotoxicity. Despite the therapy and rehabilitation no improvement was noticed—the patient continued to mobilise with a wheelchair.

## Discussion

Adhesive arachnoiditis is a rare condition and so is its description in the medical literature. The true incidence of the disease is hence unknown, and the numbers can be significantly underestimated due to the omission of subclinical cases or those describing the adhesive arachnoiditis as a cause of paraparesis in patients in whom the spinal canal stenosis has a different, undiagnosed cause [1].

The possible clinical manifestations of adhesive arachnoiditis include painful radicular syndromes with a burning pain of typical distribution of sciatic nerve and the presence of Lasegue sign, the lumbago-type pain, mobility restriction in the lumbosacral spine, neurological deficits in terms of mobility and sensation in the radicular distribution as well as the symptoms of spinal cord involvement with bladder dysfunction, pyramidal syndromes and cut superficial sensation disorders [2, 3].

The pathology of adhesive arachnoiditis arises from the fibrous invasion of the pia mater, caused by its inflammation resulting in production of fibrous tissue and adhesions, as well as a strict adherence of spinal roots to one another and/or to the thecal sac [2]. As the arachnoid has no vasculature or innervation, the healing process is very difficult, similar to that of other serous membranes like peritoneum or pleura. Constant circulation of cerebrospinal fluid makes it even more difficult by washing out the phagocytes and enzymes that prevent the formation of scar tissue [4]. In patients suffering from adhesive arachnoiditis undergoing neurosurgical procedures symptoms such as lack of pia mater pulsation, its thickening, shortage of cerebrospinal fluid, radicular oedema and fibrosis, arachnoid hyalinisation were observed [3, 4].

The aetiology of adhesive arachnoiditis is heterogenous. The most common causes include infections (bacterial, tuberculous, syphilitic), trauma (including consequences of surgical procedures), thecal sac contamination by intraspinal injection of various substances (iodine based contrast agents, corticosteroides) and spinal canal tumors [1, 3, 5, 6].

Magnetic resonance imaging (MRI) plays a crucial part in the diagnostic process of adhesive arachnoiditis. It has a sensitivity of about 92% and its specificity reaches 100% [7].

The most common MRI abnormalities are (in descending order): the presence of arachnoid cysts, clumping, thickening and displacement of nerve roots with their contrast enhancement, spinal cord swelling with T2 signal hyperintensity, arachnoid separations and spinal cord compression, displacement and anchoring, as well as atrophy of the spinal cord with formation of syrinx [1]. The radiological signs of adhesive arachnoiditis often correspond with the natural history of the disease, which is reflected in the scales used for clinical and radiological staging of adhesive arachnoiditis that are published in the literature. Those based on the MRI imaging seem to be still clinically useful, especially the three-stage-scale proposed by Delamarter [4]. The MRI imaging evidence of all three of our patients can be described as group III, standing for the most advanced stage of the disease.

The therapy of patients diagnosed with adhesive arachnoiditis is extremely difficult—there is no causative treatment, and symptoms based therapy and applied medication rarely bring expected results. Opioid and non-opioid painkillers, steroids and spinal cord stimulation can be named among the most widely used methods [2]. Surgical approach (arachnoid dissection with duroplasty) remains controversial. Its effectiveness has not been confirmed so far due to complex pathology of adhesive arachnoiditis and common postoperative recurrence or progression of the disease. Several authors reported modifications of surgical management aiming at minimising the risk of recurrence – for instance microlysis of adhesions followed by expansive duroplasty with a Gore-tex surgical membrane, expansive laminoplasty and multiple tenting sutures of Gore-tex graft as reported by Ohata et al. [8] or microdissection of thickened adherent arachnoid followed by ventriculo-subarachnoid shunt to provide flow of cerebrospinal fluid as Mitsuyama suggests [9]. Except for patient K.M., whose surgical treatment was undertaken due to a high suspicion of malignant process, the other two patients were treated conservatively. None of them recovered the strength of lower extremities. Up to date there is no agreement regarding guideline management of spinal adhesive arachnoiditis—the decisions are taken based on individual cases. Summary of case reports published since year 2000 regarding thoracic spinal adhesive arachnoiditis associated with lower limbs weakness handled either surgically or conservatively is included in Table 1.

**Table 1:** Thoracic spinal adhesive arachnoiditis: recent case reports

Case report	Patients’ data	Symptoms	Suspected aetiology	Management	Outcome
Carlswärd et al*.* [10]	Female, aged 29	Paraparesis Low back pain	Epidural anesthesia, epidural blood patch	Conservative	Walking distance of 40 m due to severe pain, wheelchair dependent
Pasoglou et al*.* [11]	Female, aged 35	Paraparesis Sensory loss at T6	Familial cases—members of a Belgian family, none of the patients had typical risk factors of adhesive arachnoiditis	T6–T5 laminectomy	Walking difficulty, sensory loss below T6 level
	Male, aged 50	Diagnosed with syringomyelia		Surgical—no data given	Died of perioperative complication
	Male, age not given	Neurologically impaired—walking difficulty No documentation available		No data	No data
	Female, aged 49	Paraparesis Loss of pain and hot–cold sensation below T3 level		Surgical, technique not given	No data
	Female, aged 45	Dorsal pain Walking difficulty Paresthesias at the thoracic dermatomes		Surgical, technique not given	No data
	Female, aged 49	Walking difficulty Urinary incontinence		Surgical, technique not given	No data
Killeen et al*.* [12]	Female, aged 27	Paraparesis Bowel and urine incontinence Sensory loss at T10 level Hydrocephalus	Spinal anesthesia	L5-S1 laminectomy, T9 laminectomy with syrinx drainage, Ventriculoperitoneal shunting	Paraparesis: wheelchair-bound, requires suprapubic catherer
Ishizaka et al*.* [13]	Female, aged 66	Minor paraparesis Gait disturbance Urine incontinence Sensory level at T10 on the left and Th6 on the right Paresthesias of the abdomen	Subarachnoid hemorrhage	Laminectomy and microlysis of the adhesions from T2 to T10 level	Could walk unassisted, paresthesias of the abdomen persisted
Takashi et al*.* [14]	Female, aged 29 (first presentation)	Paraplegia Anuresis Numbness below the chest	Spinal and epidural anesthesia	Posterior T6–T7 laminectomy with adhesiolysis	Slight recovery of motor function
	Female, aged 32 (same patient 3 years later)	Progressive worsening of locomotive function		Posterior T5–T6 laminectomy with arachnoid cyst peritoneal shunting	Able to walk unaided 3 years post operation
Kok et al. [15]	Female, aged 63	Spastic paraparesis Urinary incontinence	Subarachnoid haemorrhage complicated with hydrocephalus in both cases	Conservative	Paraparesis diminished, urinary incontinence persisting
	Female, aged 68	Pyramidal paraparesis		Conservative	Pyramidal signs diminished

What is notable in the three presented cases is the varying aetiology in each of them. In case of patient B.S.—the paraparesis coincided with a surgical procedure run in epidural block, with no history of any prior surgical interventions. This form of anaesthesia is a commonly accepted and safe procedure. Complications are exceedingly rare and, in the vast majority of cases, of transient nature, with the most common being haematomas and infections [12]. Current medical reports state that the relation between a spinal block and adhesive arachnoiditis seems questionable [8, 9, 12]. The two largest cohort studies to date, from the United Kingdom and Finland, do not link these two procedures either. Nonetheless ever since the 1950′ some reports of delayed neurological deficit following a routine spinal block do appear [16, 17]. A possible impact of the chemical agents used for anaesthesia has been postulated in the literature—caused by the anaesthetics themselves (in lab research their supra-clinical dosage caused the neuronal tissue damage [7, 18, 19]), as well as contamination of their solutions with other chemical substances, for instance phenols or detergents [7, 12, 20, 21]. However, it is worth noting that the very extravasation of blood during the spinal block procedure itself could have an impact on initialising the inflammatory process [7, 22, 23].

Another potential factor—the surgeries of the spinal canal—belong to the best recognised and most widely documented causes of adhesive arachnoiditis. This entity is commonly known as Failed Back Surgery Syndrome [2]. Not only the very surgical intervention but also the coexisting bleeding itself can contribute to the development of arachnoiditis. The case reports of adhesive arachnoiditis following subarachnoid haemorrhage led to conclusion that the presence of blood in cerebrospinal fluid can lead to an inflammatory reaction- increased intrathecal collagen synthesis, subsequent fibroproliferative process and arachnoiditis [7, 12, 24]. The case reports of arachnoiditis following epidural blood patch for treatment of postural puncture headache seem to support that hypothesis [10]. As dural breaching results in CSF contamination with blood it could initiate the inflammation as well. In Patient G.W. there seem to be at least a few factors contributing to the development of paraparesis. She did not experience any neurological deficit directly after the T7–T8 schwannoma surgery. The lower limb weakness occurred suddenly during one of the chemotherapy cycles, about 2 years after the surgery. Therefore we can presume that the aetiology of paraparesis in this case is complex—the neurotoxicity of implemented systemic treatment could also have led to the occurrence of paraparesis. We have not come across any reports of adhesive arachnoiditis following chemotherapy in the contemporary literature. However, having in mind the neurotoxic potential of chemotherapeutic agents and risk of adhesive arachnoiditis due to the toxicity of the intrathecal anaesthetics the contribution of the above risk factors cannot be neglected.

K.M. patient’s inflammatory process initiating factor seems to be most difficult to identify. On one hand it could be caused by surgery at posterior cerebral fossa with subsequent presence of blood in the cerebrospinal fluid and on the other hand it could be the consequence of the expansive process in the posterior fossa—dysregulation of cerebrospinal fluid circulation due to hydrocephalus. The recurrence of hydrocephalus after a few weeks following the operation is significant—it was then, most probably, when a secondary manifestation of adhesive arachnoiditis occurred—a similar case has been described in the literature [24].

All of described cases fall into the category of severe clinical course of illness leading to disability—none of the patients preserved the ability to ambulate, all of them ended up wheelchair-bound. What is also interesting—all of presented adhesive arachnoiditis cases involved mostly or exclusively the thoracic spine, though the literature mainly relates this pathology to the lumbosacral spine. In case of patient G.W. the location of adhesive arachnoiditis is strictly related to the T7–T8 neurosurgical procedure history. In two other two patients the triggering factor did not work in the proximity of the structures later involved in the arachnoiditis.

All of our patients were treated mostly conservatively and underwent intensive rehabilitation with no significant improvement. In one of the patients the baclofen pump implantation helped to reduce spasticity with no influence on motor skills.

## Conclusion

Adhesive arachnoiditis is a condition of various aetiology and symptomatology that can potentially lead to severe, irreversible disability. Though rare it should be considered in the differential diagnosis in patients with progressive lower limb weakness, in particular ones with a history of known risk factors. MRI imaging is crucial for the diagnostic process—it allows pointing out radiological changes typical for adhesive arachnoiditis and is strictly related to the natural history of this disease. Despite our good understanding of the nature of adhesive arachnoiditis, the contemporary medical knowledge does not provide us with any prophylactic guidelines or therapy of this rare and unpredictable disease.
